# Effects of Acute Exercise Bouts on Cardiovascular Biomarkers in Runners with Exercise-Induced Hypertension

**DOI:** 10.3390/sports13070195

**Published:** 2025-06-20

**Authors:** Young-Joo Kim, Han-Soo Park, Sang-Hyun Nam, Sang-Hoon Kim, So-Eun Lee, Jae-Hee Choi, Yong-Bum Park, Jin-Ho Yoon

**Affiliations:** 1 School of Sports Science, Sungshin Women’s University, Seoul 02844, Republic of Korea; kyj87@sungshin.ac.kr; 2 Sports Medicine Laboratory, Korean National Sport University, Seoul 05541, Republic of Korea; 302673@knsu.ac.kr; 3 Department of Plastic and Reconstructive Surgery, Sanggye Paik Hospital, Inje University, Seoul 01757, Republic of Korea; s2607@paik.ac.kr; 4 Department of Health and Rehabilitation, Osan University, Osan 18119, Republic of Korea; sportler@hanmail.net; 5 College of Wesley Creative Convergence, Hyupsung University, Hwaseong 18330, Republic of Korea; selee@omail.uhs.ac.kr; 6 Department of Sports and Life, Duksung Women’s University, Seoul 01369, Republic of Korea; cjhikki9@hanmail.net; 7 Department of Rehabilitation Medicine, Sanggye Paik Hospital, Inje University, Seoul 01757, Republic of Korea

**Keywords:** oxidative stress, endothelial dysfunction, myocardial burden, antioxidant enzymes, N-terminal pro-brain natriuretic peptide (NT-proBNP)

## Abstract

Exercise-induced hypertension (EIH) has increasingly been observed among middle-aged long-distance runners, raising concerns about cardiovascular risk. This study aimed to investigate acute changes in cardiovascular biomarkers associated with vascular inflammation, oxidative stress, antioxidant defense, endothelial function, and myocardial burden in runners with EIH. Thirty-seven middle-aged male runners (aged 40–65 years) were categorized into a normal blood pressure group (NBPG; systolic blood pressure <210 mmHg, *n* = 23) and an EIH group (EIHG; ≥210 mmHg, *n* = 14) based on maximal systolic blood pressure during a graded exercise test (GXT). Participants performed a 30 min treadmill run at 80% heart rate reserve, and blood samples were collected before and after exercise. The biomarkers analyzed included high-sensitivity C-reactive protein (hs-CRP), derivatives of reactive oxygen metabolites (d-ROMs), biological antioxidant potential (BAP), nitric oxide (NO), superoxide dismutase (SOD), and N-terminal pro-brain natriuretic peptide (NT-proBNP). The results show that the EIHG exhibited increased NT-proBNP and SOD levels, along with a reduced NO response, indicating elevated myocardial stress and impaired vasodilation. hs-CRP was positively correlated with multiple hemodynamic indices, and SOD levels were associated with maximal systolic pressure and myocardial burden. These findings highlight the need for individualized monitoring and cardiovascular risk management in runners with EIH.

## 1. Introduction

Moderate exercise enhances endothelial function, reduces cardiovascular risk, and improves cardiorespiratory fitness, contributing to a better quality of life and a lower mortality rate [1,2]. However, performing excessive exercise, such as marathon running, may lead to adverse effects, including an increased risk of atrial fibrillation [3], heightened arterial stiffness [4], and a greater prevalence of coronary artery plaques [5]. Excessive exercise can lead to persistently elevated blood pressure, which in turn increases mechanical stimulation and oxidative stress within the arteries, accelerating arteriosclerosis [6]. Additionally, high-intensity exercise elevates both volume and pressure within the left ventricle, potentially resulting in left ventricular hypertrophy [7]. In the atrium, fibrosis gradually progresses through atrial dilation, inflammation, and recovery, increasing the risk of fatal arrhythmias such as atrial fibrillation [8].

Recently, the incidence of exercise-induced hypertension (EIH), which refers to an excessive increase in blood pressure during exercise, has been reported to be high among long-distance runners [9]. Runners with EIH are known to have a higher risk of developing arrhythmia [9], a greater prevalence of coronary artery plaques [10], increased expression of cardiac markers [11], and a greater likelihood of ventricular hypertrophy compared to runners with normal blood pressure during exercise [12]. EIH is defined as a systolic blood pressure of ≥210 mmHg in men and ≥ 190 mmHg in women during maximal exercise testing [13].

In the general population, EIH is recognized as an independent risk factor for increased cardiovascular disease and mortality [14,15]. While there are no longitudinal studies linking EIH to higher mortality rates in long-distance runners, concerns persist that runners with EIH may face a heightened risk of sudden cardiac death during exercise or competition [16]. The excessive increase in blood pressure during exercise in EIH runners may be attributed to increased afterload, resulting from endothelial dysfunction in peripheral blood vessels induced by excessive exercise [17]. This endothelial dysfunction, along with increased arterial stiffness, is known to enhance sympathetic nervous system activation, further exacerbating the hypertensive response during exercise [15,17]. Although pharmacological treatments such as angiotensin II receptor blockers (ARBs) have shown efficacy in managing EIH, no official treatment guidelines have yet been established [18]. Prolonged exposure to EIH during exercise or competition may lead to a cumulative effect, gradually increasing the likelihood of negative impacts on the heart and blood vessels.

This study aims to provide initial evidence on how a single bout of exercise affects biomarkers of vascular inflammation (hs-CRP), oxidative stress (d-ROMs), antioxidant potential (BAP), antioxidant enzyme activity (SOD), vasodilatory function (NO), and myocardial burden (NT-proBNP) in runners with exercise-induced hypertension (EIH).

## 2. Materials and Methods

### 2.1. Participants and Study Protocol

**Figure 1:**
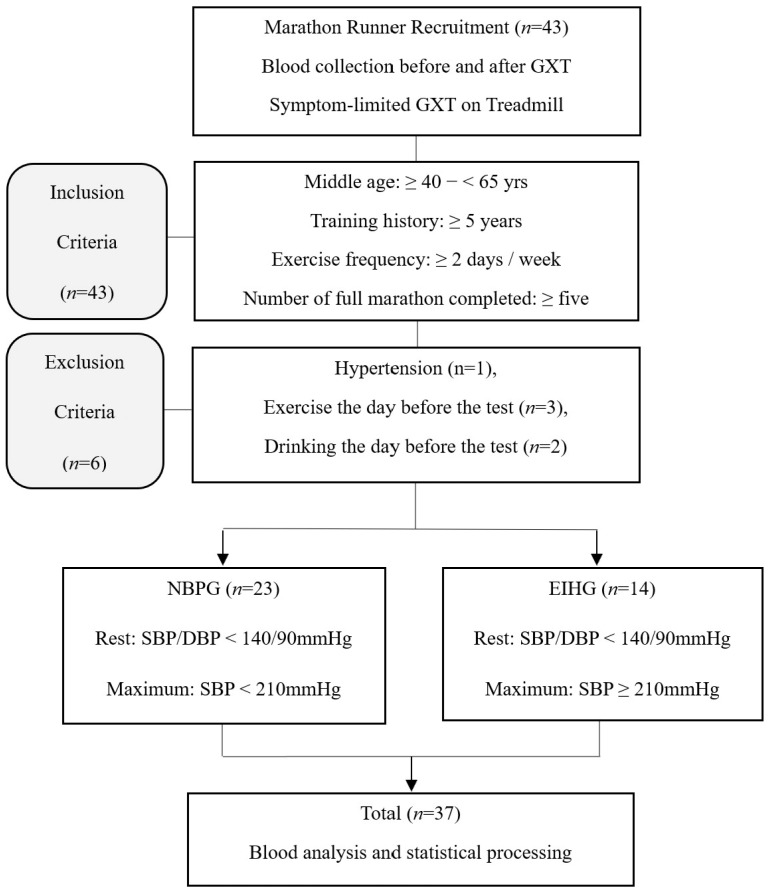
The flow chart of the study procedure. GXT: graded exercise testing, NBPG: normal blood pressure group, EIHG: exercise-induced hypertension group.

As outlined in Figure 1, participants were restricted to middle-aged individuals between the ages of 40 and 65 years. Inclusion criteria required a minimum training history of five years, a training frequency of at least twice per week, and completion of at least five marathons. Of the 43 individuals who applied, 37 completed the full GXT protocol. Although one participant reported lower limb pain, he successfully reached maximal effort during the test and was therefore included in the final analysis. One participant was excluded due to previously undiagnosed hypertension identified during pre-test screening. Additionally, two participants were excluded for engaging in exercise within 24 h prior to testing, and three for alcohol consumption during the same period.

The participants were categorized into two groups based on their maximal systolic blood pressure (SBPmax) during a graded exercise test (GXT); individuals with an SBPmax of <210 mmHg were assigned to the normal blood pressure group (NBPG), and those with SBPmax ≥210 mmHg were assigned to the exercise-induced hypertension group (EIHG), following established criteria [13]. All participants completed a 30 min treadmill run at 80% heart rate reserve (HRR). Blood samples were collected immediately before the GXT and immediately after the exercise session.

Exercise intensity for each participant was individually prescribed based on the Karvonen formula [19], using heart rate reserve (HRR) calculated from the graded exercise test. The target heart rate was determined using the following equation:

Target HR = [(HRmax − HRrest) × 0.80] + HRrest

This study was approved by the Institutional Review Board of Korea National Sport University (Approval No: 20230921-090) and was conducted in accordance with the 1975 Declaration of Helsinki.

### 2.2. Graded Exercise Test

A graded exercise test (GXT) was conducted to evaluate the participants’ hemodynamic responses and cardiorespiratory fitness at rest and during exercise. The Bruce protocol was utilized during the test using a treadmill (T170DE, h/p/cosmos, Nussdorf-Traunstein, Germany), with each stage lasting 3 min. At 2 min and 30 s of each stage, heart rate, electrocardiogram (CH2000, Cambridge Heart, Bedford, MA, USA), blood pressure (Tango+, SusnTech, Morrisville, NC, USA), respiratory gas analysis (Quark CPET, Cosmed, Rome, Italy), and rating of perceived exertion (Borg scale) were measured. A high-performance microphone was placed in direct contact with the brachial artery to obtain accurate blood pressure readings, while the examiner utilized headphones to ensure precise measurements. All procedures for the GXT were conducted under American College of Cardiology and American Heart Association guidelines, including criteria for exercise contraindications and termination [20]. The detailed stage-wise structure and measurement schedule of the GXT protocol are presented in Table 1.

### 2.3. Blood Sampling and Analysis

Blood samples were collected from the brachial vein in a fasting state using serum separator tubes (BD Microtainer^®^ SST™, BD, Franklin Lakes, NJ, USA). After collection, the samples were centrifuged at 3000 rpm for 10 min at 4 °C. The separated serum was aliquoted and stored at −80 °C until further analysis.

High-sensitivity C-reactive protein (hs-CRP), an inflammatory biomarker, was measured using the particle-enhanced immunoturbidimetric method on a Cobas 6000/C501 analyzer (Roche Diagnostics, Basel, Switzerland). The coefficient of variation (CV) for hs-CRP was less than 5%.

Oxidative stress was assessed using derivatives of reactive oxygen metabolites (d-ROMs), measured by colorimetric analysis with specific reagents (DIA CRON Srl, Grosseto, Italy). Biological antioxidant potential (BAP), representing antioxidant capacity, was also evaluated via the same colorimetric method using the BAP kit (DIA CRON Srl).

Nitric oxide (NO), a marker of vascular endothelial function, was determined using a colorimetric assay with reagents from R&D Systems (Minneapolis, MN, USA), and absorbance was measured using a VersaMax Microplate Reader (Molecular Devices, San Jose, CA, USA).

Superoxide dismutase (SOD), an endogenous antioxidant enzyme, was analyzed using the Cayman SOD Assay Kit (Cayman Chemical, Ann Arbor, MI, USA), with absorbance also measured on the VersaMax Microplate Reader.

N-terminal pro-brain natriuretic peptide (NT-proBNP), an index of myocardial stress, was quantified via electrochemiluminescence immunoassay using Roche reagents on a Cobas 8000/e602 analyzer (Roche Diagnostics, Basel, Switzerland). The CV for NT-proBNP was also below 5%.

The selected biochemical markers have been validated in previous studies for evaluating cardiovascular function, inflammation, oxidative stress, and myocardial load in response to exercise. hs-CRP is a widely used marker of systemic inflammation and cardiovascular risk, responsive to acute and chronic exercise stimuli [11,21]. d-ROMs and BAP are established indicators of oxidative balance and have shown prognostic value in cardiovascular health [22,23]. NO reflects endothelial function and shear-stress-induced vasodilation [24], while SOD is a key antioxidant enzyme elevated in response to exercise-induced reactive oxygen species [25,26]. NT-proBNP is a clinically established marker of myocardial stress and is sensitive to acute hemodynamic changes during exercise [11,27].

### 2.4. Statistical Analysis

Statistical analysis was conducted using SPSS Statistics version 21 (IBM Corporation, Armonk, NY, USA), and all measured values were expressed as mean ± standard deviation. An independent *t*-test was performed to compare the differences in demographic, hemodynamic, and cardiorespiratory fitness characteristics between the two groups. A two-way repeated ANOVA was used to examine the interaction effects (time × group) before and after exercise. If a significant interaction effect was found, simple main effects analyses were performed to assess between-group differences at each time point and within-group differences over time. The Pearson correlation coefficient was calculated to assess the correlation between rest blood analysis values, blood pressure, and myocardial burden ratio. Statistical significance was set at *p* < 0.05.

## 3. Results

### 3.1. Baseline Characteristics

A total of 37 participants were included in the final analysis, with 23 assigned to the NBPG and 14 to the EIHG based on their SBPmax during GXT. Table 2 summarizes the demographic, hemodynamic, and cardiorespiratory fitness characteristics of the two groups.

No significant differences were observed between the groups in age, height, weight, or body mass index (BMI). HRrest also did not differ significantly. However, HRmax was significantly higher in NBPG than in EIHG (*p* < 0.05). SBPmax and DBPmax were significantly higher in EIHG compared to NBPG (*p* < 0.05).

There were no significant group differences in VO_2_max, total exercise duration, number of marathons completed, weekly exercise time, training intensity, or marathon time. However, the NBPG had a significantly longer training history than the EIHG (*p* < 0.05).

Regarding post-exercise recovery, there were no significant differences in heart rate recovery between the groups, but both systolic and diastolic blood pressure recovery (RSBP and RDBP) values were significantly higher in EIHG at all measured time points (*p* < 0.05).

### 3.2. Blood Sampling and Analysis

Table 3 compares inflammatory response, reactive oxygen species concentration, and antioxidant capacity between the two groups. All values in parentheses indicate pre- and post-exercise measurements.

The inflammatory marker hs-CRP showed minimal changes in both groups: NBPG (0.55 ± 0.40 vs. 0.56 ± 0.41) and EIHG (0.86 ± 0.61 vs. 0.89 ± 0.64). The oxidative stress marker d-ROMs increased slightly in both groups—NBPG (297.0 ± 62.2 vs. 311.3 ± 56.6), EIHG (278.4 ± 66.0 vs. 315.5 ± 76.9). Antioxidant capacity, as measured by BAP, also increased post-exercise—NBPG (1840.5 ± 268.4 vs. 2108.9 ± 270.4), EIHG (1772.3 ± 334.4 vs. 2097.8 ± 330.5). However, no significant time × group interaction effects were observed for hs-CRP, d-ROMs, or BAP (*p* = 0.240, *p* = 0.279, *p* = 0.567, respectively).

**Figure 2:**
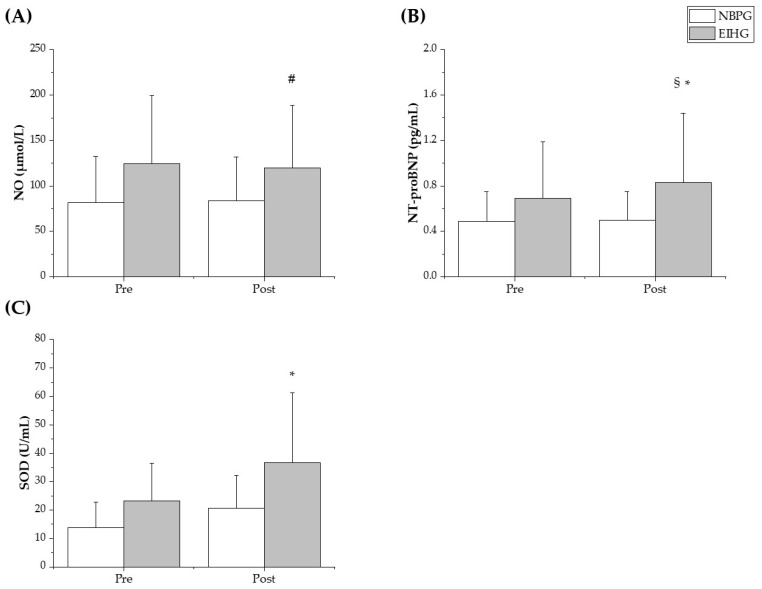
Changes in cardiovascular biomarkers before and after exercise in the normal blood pressure group (NBPG, white bars) and the exercise-induced hypertension group (EIHG, gray bars). (**A**) Nitric oxide (NO, μmol/L). A significant group × time interaction was observed (*p* = 0.038), although within-group differences were not statistically significant. The EIHG showed a trend towards decreased NO post-exercise. # *p* < 0.1 vs. pre-exercise (EIHG only; trend-level significance). (**B**) N-terminal pro-brain natriuretic peptide (NT-proBNP, pg/mL). NT-proBNP levels significantly increased after exercise in both groups, with a higher post-exercise value in the EIHG. A significant group × time interaction was observed (*p* < 0.05). * *p* < 0.05 vs. pre-exercise; § *p* < 0.05 vs. NBPG (post-exercise only). (**C**) Superoxide dismutase (SOD, U/mL). SOD levels significantly increased only in the EIHG post-exercise. A significant group × time interaction was found (*p* < 0.05). * *p* < 0.05 vs. pre-exercise (EIHG only).

Regarding antioxidant enzyme activity and myocardial burden, significant group differences were observed. NO levels showed minimal changes—NBPG (81.7 ± 50.9 vs. 83.4 ± 48.3) and EIHG (124.5 ± 74.9 vs. 119.5 ± 69.2)—however, a significant group × time interaction was detected (*p* < 0.05) (Figure 2A). NT-proBNP, a marker of myocardial burden, increased markedly after exercise—NBPG (13.9 ± 8.8 vs. 20.7 ± 11.4), EIHG (23.3 ± 13.2 vs. 36.8 ± 24.4)—also demonstrating a significant interaction effect (*p* < 0.05) (Figure 2B). Similarly, SOD activity increased in both groups, particularly in the EIHG—NBPG (0.49 ± 0.26 vs. 0.50 ± 0.25), EIHG (0.69 ± 0.50 vs. 0.83 ± 0.61)—again with a significant group × time interaction (*p* < 0.05) (Figure 2C).

### 3.3. Correlation Between Biomarkers and Hemodynamic Parameters

Table 4 presents the correlations between resting hs-CRP and SOD values and hemodynamic indices. Resting hs-CRP showed significant positive correlations with RSBP (r = 0.467, *p* < 0.05), RDBP (r = 0.392, *p* < 0.05), SBPmax (r = 0.385, *p* < 0.05), MDBP (r = 0.408, *p* < 0.05), and MRPP (r = 0.338, *p* < 0.05). In contrast, SOD was not significantly correlated with RSBP (r = –0.34), RDBP (r = 0.135), or MDBP (r = 0.080), but showed significant correlations with SBPmax (r = 0.372, *p* < 0.05) and MRPP (r = 0.377, *p* < 0.05).

## 4. Discussion

This study investigated the responses of oxidative stress, antioxidant markers, and cardiovascular biomarkers in runners with EIH following 30 min of high-intensity running at 80% HRR immediately after a maximal exercise stress test.

First, the inflammatory response (hs-CRP), reactive oxygen species (d-ROMs), and antioxidant capacity (BAP) significantly increased after a single bout of exercise in both groups; however, no significant interaction effect was observed. Hs-CRP, a cardiovascular risk assessment marker used in patients with coronary artery disease, decreases with regular exercise [21]. However, in EIH runners, hs-CRP increases immediately after excessive exercise, such as a 100 km ultramarathon [27], whereas no significant change has been reported following a marathon race [11].

In this study, we found no significant change following a single bout of exercise, suggesting that the elevation of this inflammatory marker occurs only during prolonged and extreme exercise, such as an ultramarathon, in EIH runners. The relationship between hs-CRP and hemodynamic responses was examined, revealing a significant positive correlation not only with resting blood pressure but also with the blood pressure response to maximal exercise and maximal myocardial burden. These findings highlight the importance of blood pressure management in runners. d-ROMs and BAP are markers for assessing cardiovascular disease risk [22,23], with a d-ROMs level of 395 or higher serving as a critical threshold for increased cardiovascular mortality risk [22]. In this study, all values remained within the normal range, and both groups exhibited a similar increase in d-ROMs levels after a single bout of exercise.

One of the key findings of our study is that even a single bout of exercise placed a significant myocardial burden on runners with EIH. This effect is primarily driven by increased afterload due to impaired peripheral arterial dilation, resulting in elevated systolic blood pressure during exercise, as well-documented in previous studies [17]. In this study, EIH runners exhibited a decrease in NO levels rather than an increase immediately after a single bout of exercise, indicating impaired vasodilation (Figure 2A). Kim et al. [28] reported that NO levels decreased more in EIH runners than in NBPG after completing a Maximal GXT. Additionally, Kim et al. [29] found that NO did not significantly increase even immediately after a 100 km ultramarathon in EIH runners. NO is typically expressed in response to shear stress on endothelial cells induced by exercise [24], but in runners with EIH, NO fails to increase appropriately. The attenuated NO response observed in the EIH group may be partially explained by endothelial nitric oxide synthase (eNOS) dysregulation, which compromises endothelial-dependent vasodilation, particularly under conditions of arterial stiffness and elevated shear stress [17,24]. Moreover, heightened oxidative stress during intense exercise increases the production of reactive oxygen species (ROS), which rapidly react with NO to form peroxynitrite, thereby further reducing its bioavailability and vascular effects [6,25,26]. These molecular pathways suggest that chronic vascular stress in EIH runners may disrupt NO-mediated vasodilation both at the enzymatic and redox levels. This inadequate NO response contributes to elevated blood pressure due to increased afterload, subsequently leading to an increase in NT-proBNP, a marker of left ventricular volume pressure [27]. In this study, a single bout of exercise led to a significant increase in NT-proBNP levels in EIH runners (Figure 2B). Previous studies have reported that, immediately after a marathon and a 100 km ultramarathon, EIH runners exhibited significantly higher NT-proBNP levels compared to those with normal blood pressure during exercise [11,27]. The findings of this study, along with previous research, suggest that EIH runners are exposed to relatively higher myocardial stress not only during long-distance races but also following a single bout of exercise. Runners with EIH experience chronic elevations in systolic blood pressure during exercise, leading to increased myocardial burden and the development of myocardial hypertrophy [12]. This suggests that EIH runners undergo a relatively higher exercise intensity due to elevated blood pressure and increased myocardial burden, even when training at a similar heart rate intensity as runners with normal blood pressure during exercise.

Further supporting this finding, our study observed an increase in the antioxidant enzyme SOD in EIH runners (Figure 2C). It is widely recognized that SOD levels rise as exercise intensity increases [25]. The relative increase in SOD after a single bout of exercise in EIH runners can be explained as an antioxidant defense mechanism to protect against oxidative stress during high-intensity exercise [26]. More interestingly, resting SOD levels showed a positive correlation with SBPmax and MRPP, indicating that higher blood pressure and myocardial burden during exercise are associated with elevated antioxidant enzyme activity. These findings suggest that the increase in SOD in EIH runners is a protective response to oxidative stress. Therefore, strategies to regulate excessive blood pressure elevation during exercise should be explored. We confirmed that even a single bout of exercise in EIH runners led to an increased myocardial burden due to impaired vasodilation, along with a compensatory increase in SOD as a protective response to elevated oxidative stress. Therefore, if EIH runners are chronically exposed to single-bout exercises outside of competition, the risk of cardiovascular disease cannot be ruled out, highlighting the need for long-term follow-up studies.

These findings, although derived from acute exercise responses, may carry potential prognostic implications. The repeated elevation of NT-proBNP and blunted NO response observed in EIH runners could, over time, contribute to cardiac remodeling [12] and increased cardiovascular risk [10], especially in the absence of clinical symptoms. Previous studies have suggested that persistently elevated exercise-induced blood pressure is associated with adverse long-term outcomes, including left ventricular hypertrophy and cardiovascular events [9,13,14,15]. Therefore, these biomarkers may serve as early indicators for identifying high-risk individuals who require longitudinal monitoring or targeted interventions.

This study has several limitations. First, the study exclusively recruited middle-aged male runners (aged 40–65 years), which significantly limits the generalizability of the findings to other age groups or to female athletes. Second, although lifestyle behaviors were assessed via self-reported questionnaires and interviews, no objective controls (e.g., standardized nutrition intake, sleep quality tracking, or activity monitoring) were implemented. As a result, unmeasured day-to-day variations in these confounding variables may have influenced physiological responses. Third, while the 30 min exercise duration was consistent between the two groups, the total exercise time varied among individuals due to differences in the duration of the maximal exercise stress test conducted beforehand. Although this did not result in a statistically significant difference, achieving complete uniformity was not feasible. Fourth, the combined use of a maximal GXT and a submaximal 30 min treadmill run on the same day may have amplified acute physiological responses. Although the GXT was followed by a short recovery period, initiating a subsequent run at 80% HRR immediately afterward could have introduced additional cardiovascular strain. This combined protocol was chosen to simulate a continuous training challenge, but we acknowledge that separating these sessions may be preferable in future studies to reduce physiological variability and isolate specific effects.

## 5. Conclusions

In EIH runners, a single bout of high-intensity exercise resulted in increased myocardial burden markers due to elevated afterload caused by impaired arterial vasodilation. This condition led to heightened expression of antioxidant enzymes as a compensatory response to increased oxidative stress from exercising at a higher intensity. If this response becomes chronic, concerns regarding potential adverse effects on cardiovascular and cerebrovascular health may arise, underscoring the need for further longitudinal studies.

## Figures and Tables

**Table 1:** Graded exercise test protocol used in this study.

Stage	Time (min)	Speed (mph)	Grade (%)	Measurement	Description
Rest	0–5	-	-	HR, SBP, DBP, RPP	5 min seated rest prior to exercise
Stage 1	0–3	1.7	10	HR, SBP, DBP, RPE, ECG	Measurements taken at 2 min 30 s
Stage 2	3–6	2.5	12	HR, SBP, DBP, RPE, ECG	Measurements taken at 2 min 30 s
Stage 3	6–9	3.4	14	HR, SBP, DBP, RPE, ECG	Measurements taken at 2 min 30 s
Stage 4	9–12	4.2	16	HR, SBP, DBP, RPE, ECG	Measurements every 30 s
Stage 5	12–15	5.0	18	HR, SBP, DBP, RPE, ECG	Measurements every 30 s
Stage 6	15–18	5.5	20	HR, SBP, DBP, RPE, ECG	Measurements every 30 s
Stage 7	18–21	6.0	22	HR, SBP, DBP, RPE, ECG	Measurements every 30 s
Recovery	21–24	1.7	0	HR, SBP, DBP	Recovery monitoring during treadmill walking

GXT: graded exercise test, Reco: recovery, RPE: rating of perceived exertion, SBP: systolic blood pressure, DBP: diastolic blood pressure, HR: heart rate, ECG: electrocardiography. Note: Blood pressure was monitored using an amplified stethoscope with a high-sensitivity microphone on the brachial artery. Protocol: Bruce protocol, 3 min per stage. Testing terminated per ACC/AHA guidelines.

**Table 2:** Characteristics of demographic, hemodynamic, and cardiorespiratory fitness.

	NBPG (*n* = 23)	EIHG (*n* = 14)	*p* Value
General characteristics			
Age, years	57.2 ± 6.7	59.4 ± 6.2	0.327
Height, cm	169.3 ± 4.8	173.0 ± 8.4	0.100
Weight, kg	65.1 ± 5.6	70.3 ± 11.7	0.139
BMI, m·Ht-2	23.7 ± 5.0	23.3 ± 1.8	0.739
Hemodynamic characteristics			
HRrest, BPM	56.4 ± 5.5	52.3 ± 9.1	0.142
HRmax, BPM	164.7 ± 10.5	155.2 ± 12.8	0.028
SBPrest, mmHg	125.7 ± 9.2	127.3 ± 11.5	0.634
SBPmax, mmHg	182.6 ± 13.2	219.5 ± 13.5	<0.001
DBPrest, mmHg	79.9 ± 5.9	79.9 ± 6.8	0.990
DBPmax, mmHg	93.3 ± 7.6	101.6 ± 5.8	0.001
HRrec (1 min), BPM	133.5 ± 16.1	132.6 ± 21.2	0.888
HRrec (2 min), BPM	112.2 ± 11.2	113.8 ± 21.2	0.761
HRrec (3 min), BPM	98.2 ± 12.2	102.1 ± 10.6	0.530
SBPR (1 min), mmHg	180.8 ± 14.1	216.6 ± 7.9	<0.001
SBPR (2 min), mmHg	173.4 ± 16.0	205.7 ± 8.8	<0.001
SBPR (3 min), mmHg	165.1 ± 15.7	192.8 ± 10.3	<0.001
DBPR (1 min), mmHg	91.8 ± 7.5	100.2 ± 7.6	0.003
DBPR (2 min), mmHg	87.2 ± 10.3	96.0 ± 6.8	0.007
DBPR (3 min), mmHg	84.4 ± 10.2	92.2 ± 7.2	0.019
Exercise data			
Training experience (years)	19.0 ± 6.3	15.0 ± 4.7	0.044
Marathons completed (number)	78.9 ± 84.6	94.5 ± 100.5	0.556
Exercise time (min/day)	87.4 ± 35.0	72.8 ± 23.0	0.191
Exercise intensity (Borg’s RPE scale)	13.1 ± 1.8	13.1 ± 1.4	0.957
Marathon time (min)	218.2 ± 31.3	234.8 ± 38.2	0.160
Physical performance			
VO_2_ max (kg/mL/min)	51.3 ± 5.9	47.1 ± 8.8	0.094
Total exercise (time)	808.0 ± 111.1	732.1 ± 94.3	0.041

Data are presented as mean ± standard deviation. NBPG: normal blood pressure group, EIHG: exercise-induced hypertension group, BMI: body mass index, BPM: beat per minute, HR: heart rate, SBP: systolic blood pressure, DBP: diastolic blood pressure, HRrec: heat rate recovery, SBPR: systolic blood pressure recovery, DBPR: diastolic blood pressure recovery, RPE: rating of perceived exertion.

**Table 3:** Changes in hs-CRP, d-ROMs, and BAP before and after exercise and GXT.

Variable	Group	Pre	Post	*p* Value
hs-CRP (mg/L)	NBPG EIHG	0.55 ± 0.40 0.86 ± 0.61	0.56 ± 0.41 0.89 ± 0.64	0.022 ^a^ 0.076 ^b^ 0.240 ^c^
d-ROMs (U.CARR)	NBPG EIHG	297.0 ± 62.2 278.4 ± 66.0	311.3 ± 56.6 315.5 ± 76.9	0.019 ^a^ 0.704 ^b^ 0.279 ^c^
BAP (μmol/L)	NBPG EIHG	1840.5 ± 268.4 1772.3 ± 334.4	2108.9 ± 270.4 * 2097.8 ± 330.5 *	<0.001 ^a^ 0.650 ^b^ 0.567 ^c^

Data are presented as mean ± standard deviation. NBPG: normal blood pressure group, EIHG: exercise-induced hypertension group, hs-CRP: high sensitive C-reactive protein, d-ROMs: derivatives of reactive oxygen metabolites, BAP: biological antioxidant potential, ^a^: time, ^b^: group, ^c^: time × group, *: significantly different from the pre at *p* < 0.05.

**Table 4:** Correlation of hs-CRP and SOD to hemodynamic responses.

	RSBP (mmHg)	RDBP (mmHg)	MSBP (mmHg)	MDBP (mmHg)	MRPP (HR × SBP)
hs-CRP (mg/dL)	r = 0.467 *	r = 0.392 *	r = 0.385 *	r = 0.408 *	r = 0.338 *
SOD	r = −0.034	r = 0.135	r = 0.372 *	r = 0.080	r = 0.377 *

hs-CRP: high sensitivity C-reactive protein, SOD: super oxide dismutase, RSBP: resting systolic blood pressure, RDBP: resting diastolic blood pressure, MSBP: maximal systolic blood pressure, MDBP: maximal diastolic blood pressure, MRPP: maximal rate pressure product, *: *p* < 0.05.

## Data Availability

The data that support the findings of this study are available from the corresponding author upon reasonable request.

## Abbreviations

The following abbreviations are used in this manuscript:

EIH	Exercise-Induced Hypertension
NBPG	Normal Blood Pressure Group
EIHG	Exercise-Induced Hypertension Group
GXT	Graded Exercise Test
HRR	Heart Rate Reserve
hs-CRP	High-Sensitivity C-Reactive Protein
d-ROMs	Derivatives of Reactive Oxygen Metabolites
BAP	Biological Antioxidant Potential
NO	Nitric Oxide
SOD	Superoxide Dismutase
NT-proBNP	N-terminal pro-brain Natriuretic Peptide
SBPmax	Maximal Systolic Blood Pressure
DBPmax	Maximal Diastolic Blood Pressure
HRrest	Resting Heart Rate
HRmax	Maximal Heart Rate
VO_2_max	Maximal Oxygen Consumption
RSBP	Recovery Systolic Blood Pressure
RDBP	Recovery Diastolic Blood Pressure
MDBP	Mean Diastolic Blood Pressure
MRPP	Maximal Rate Pressure Product
